# Mechanical Metamaterials Gyro-Structure Piezoelectric Nanogenerators for Energy Harvesting under Quasi-Static Excitations in Ocean Engineering

**DOI:** 10.1021/acsomega.1c01687

**Published:** 2021-05-28

**Authors:** Pengcheng Jiao, Yang Yang, KingJames Idala Egbe, Zhiguo He, Yingtien Lin

**Affiliations:** † Hainan Institute of Zhejiang University, Sanya 572025, Hainan, China; ‡ Institute of Port, Coastal and Offshore Engineering, Ocean College, Zhejiang University, Zhoushan 3216021, Zhejiang, China; § Engineering Research Center of Oceanic Sensing Technology and Equipment, Ministry of Education, Zhejiang University, Hangzhou 310000, Zhejiang, China

## Abstract

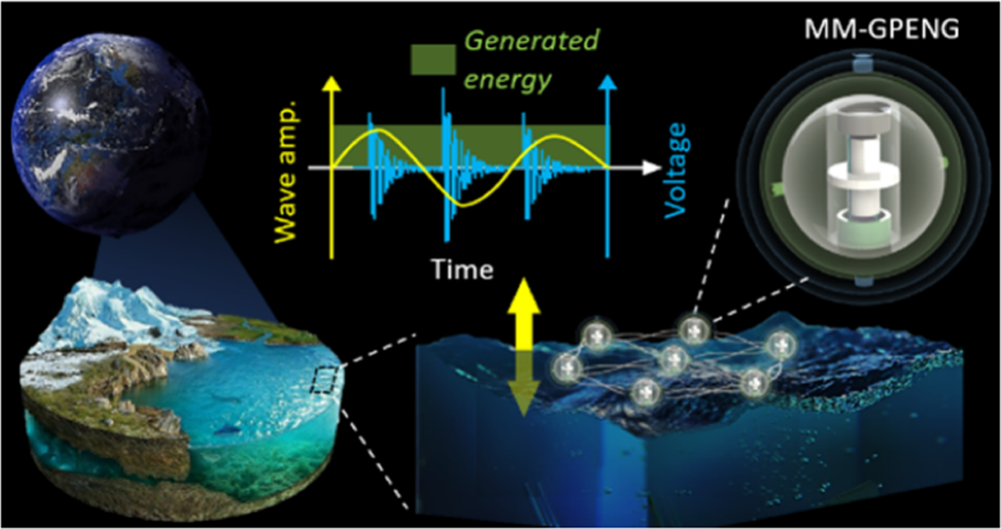

In this study, we develop the mechanical metamaterial-enabled piezoelectric nanogenerators in the gyro-structure, which is reported as a novel green energy solution to generate electrical power under quasi-static excitations (i.e., <1 Hz) such as in the ocean environment. The plate-like mechanical metamaterials are designed with a hexagonal corrugation to improve their mechanical characteristics (i.e., effective bending stiffnesses), and the piezoelectric trips are bonded to the metaplates. The piezo-metaplates are placed in the sliding cells to obtain the post-buckling response for energy harvesting under low-frequency ocean motions. The corrugated mechanical metamaterials are fabricated using the three-dimensional additive manufacturing technique and are bonded with polyvinylidene fluoride strips, and the nanogenerator samples are investigated under the quasi-static loading. Theoretical and numerical models are developed to obtain the electrical power, and satisfactory agreements are observed. Optimization is conducted to maximize the generated electrical power with respect to the geometric consideration (i.e., changing the corrugation pattern of the mechanical metamaterials) and the material consideration (i.e., changing the mechanical metamaterials to anisotropic). In the end, we consider the piezoelectric nanogenerators as a potential green solution for the energy issues in other fields.

## 1 Introduction

Traditional energy sources, for example, disposable batteries, suffer from severe limitations given the difficulty, if not impossible, of regular replacement.^1^ It is desirable to develop new energy solutions that continuously generate reliable electrical power. Classifying on the basis of converting power *from* whom/what, energy harvesting sources can be characterized into (1) human motion and (2) environment.^2^ Given the typically remote workplace in the marine environment, energy harvesting in ocean engineering has mainly been conducted on various ambient sources such as ocean waves,^3,4^ wind energy,^5^ tide energy, or marine chemical energy.^6^ In particular, blue energy from ocean waves has recently opened promising avenues for efficiently powering small-scale and low-power electronic devices for multifunctional applications.^7^

The lack of continuously reliable blue energy is one of the most severe challenges in ocean engineering, and thus, research efforts have been dedicated to developing energy harvesting techniques to address the power issue. Piezoelectric materials are extensively deployed to generate energy from their environments.^8,9^ Taking advantage of the mechanical-to-electrical materials, energy systems (namely, resonant harvesters) are developed to convert vibrations to electricity. The effectiveness of vibration-based energy harvesting solutions was investigated for low-frequency ambient sources,^10^ which indicated that excitation critically affects the efficiency of nonlinear harvesters. On the other hand, many application scenarios (e.g., ocean waves) cannot provide high ambient frequencies. Consequently, piezo-based energy harvesters typically suffer from critical limits such as ineffective output power due to the narrow range of response frequency for piezoelectric materials. Because an environment typically exhibits significantly small vibration motions at very low frequency, different approaches have been proposed to increase the quasi-static ocean waves to relatively high-frequency accelerations such that the piezoelectric materials can be effectively activated. For example, piezoelectric beams were investigated under conditions, including cantilevered^11^ and clamped–clamped^12^ boundary conditions, or in bistable/multistable systems.^13^ Taylor et al.^14^ developed the eel-like energy harvester to generate electrical power through long polyvinylidene fluoride (PVDF) strips that were directly triggered by ocean waves (i.e., without increasing frequency). Chiba et al.^15^ developed the ocean generators using the dielectric elastomer artificial muscle based on the changes in capacitive energy of deformable dielectrics. Mass-spring systems^16^ and magneto-mechanical systems^3^ were reported to convert ocean waves through the piezoelectric effect. Studies have further been conducted on composite piezoelectric materials for specific applications, for example, the energy-harvesting devices via ocean waves and sunlight,^17−19^ raindrops,^20,21^ heartbeat,^22,23^ and water,^24^ and the self-powered monitoring devices via vibration^25,26^ and rotation.^27^

Architected metastructures, typically known as mechanical metamaterials (MM), have attracted research interests due to their preponderant mechanical characteristics that are rarely obtained from traditional structures. Taking advantage of periodically repeated unit cells, the engineered structures have been reported in the literature for their promising mechanical behaviors. Keeping the material and overall geometry the same, metastructures expand their mechanical response due to the unit cells. Studies have been carried out to exploit the promising behaviors of MMs using *geometric* strategies such as lattice and auxetic structures^28,29^ or origami and kirigami,^30−33^ and *material* strategies such as 3D printed multimaterials^34^ or graphene reinforcements.^35^ Using these strategies, the overall MMs exhibit a significant geometric nonlinearity while the materials are in a linear elastic regime, which leads to desirable mechanical performance such as complete recovery from large strains in compression,^36,37^ tension,^38^ rotation,^39^ bending/buckling,^40^ ultralight with high stiffness,^36^ and programmable properties, for example, bulk modulus or mass density.^41^

Recently, studies have been reported on maneuvering ambient excitations through postbuckling responses for piezoelectric-based energy harvesters.^42,43^ Placing axially loaded plate-like MMs between the bilateral constraints, MM-PENG was reported to convert the quasi-static axial displacement into postbuckling snap-throughs and generate electrical power.^44^ Activated by quasi-static ambient excitations, the bi-walled piezo-MM performed instable mode transitions (i.e., postbuckling behavior), which efficiently prompted the piezo attachment and, eventually, converted low-frequency mechanical input into electrical output. The mechanism has later been integrated with piezo-floating-gate sensors to allow for the self-powered sensing and health monitoring in civil infrastructures.^44−46^ However, one of the most severe obstacles in the MM-enhanced PENG is *how to* maximize the harvested energy in the complex marine environment, that is, maximizing the harvested electrical power significantly depends on the bi-walled beams. Although buckling and postbuckling behaviors have been investigated in the previous studies,^47,48^ the postbuckling response has yet to be accurately controlled and predicted. As a consequence, it is desirable to program the postbuckling behavior of the bi-walled piezo-MM to trigger the attached piezoelectric strips for the optimal electrical power.

Here, we propose the MM gyro-structure piezoelectric nanogenerators (MM-GPENGs) for energy harvesting from ocean waves. The piezo-MM plates are constrained by sliding cells, which are designed in the gyro-structures to universally generate electrical power from the ocean waves in arbitrary directions. The rest of the paper is organized as follows: Section 2 introduces the main results including the design principles, fabrication, and testing of the MM-GPENG subjected to ocean motions. The MM plates were fabricated using 3D printing and are attached by piezo strips. The theoretical and numerical models are developed to validate the experimental output, and satisfactory agreements are obtained. Section 3 presents the optimization of the MM-GPENG with respect to the geometric and material considerations. Section 4 concludes the main findings of the study.

## 2 Results

### 2.1 Principles for Energy Harvesting in the Ocean Environment

**Figure 1:**
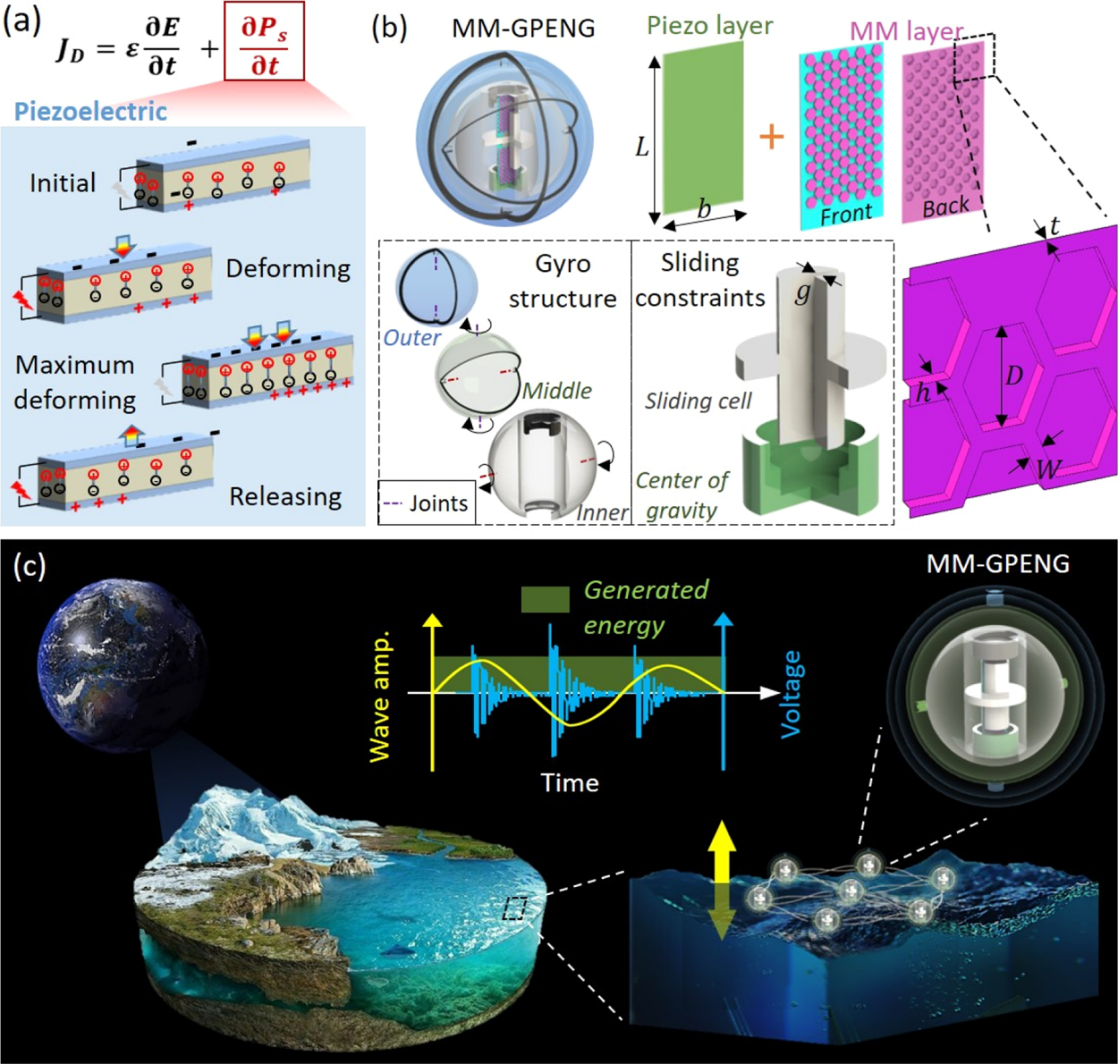
Design components and principle of the MM-GPENG. (a) Principle of piezoelectric effect. (b) Components of the MM-GPENG comprising the outer, middle, and inner spheres in the gyro-structures. Hexagonal MM plates are attached with the planar piezo layer, which are subjected to the sliding cell. The gravity center is designed such that the piezo-MM plates and sliding cell are maintained in the vertical direction under arbitrary ocean waves. (c) Illustration of the design concept of the MM-GPENG for energy harvesting in the ocean environment. The MM-GPENG is assembled into the energy harvesting net for higher energy output.

Figure 1 illustrates the MM-GPENG expanded from that reported in ref (44) to harvest electrical energy in the ocean environment. Figure 1a demonstrates the principle of the piezoelectric effect, which can be demonstrated in the metal–insulator–metal sandwich structures consisting of the insulating piezoelectric layer between the two metal electrode layers. Initially, the cations and anions are overlapped and no polarization exists in the piezoelectric materials. Next, the volumes of the piezoelectric materials are reduced, and negative strains are generated by deformation. The cations and anions are separated to create the electrical dipoles and electric dipole moments change, and thus, the piezoelectric potential is generated between the electrodes. Connecting the electrodes with the external excitations, the piezoelectric potential leads the electrons to flow in the external circuit to partially screen the potential and obtain an updated equilibrium state. Therefore, the mechanical energy that resulted in the deformation is converted into electrical energy by a new balance. When the two conductive electrodes are deformed to be completely contacted, the maximum pressed state is achieved with the highest polarization density. Finally, the electrons flow back to reach a new equilibrium condition when the external force is released in the short circuit condition. Figure 1b details the design of the MM-GPENG. The MM plates are corrugated with the hexagonal pattern and are bonded with the piezo layer. The piezo-MMs are placed in the sliding cell that is designed to convert the ocean waves into an axial displacement through inertia. The gyro-structure comprised the inner, middle, and outer spheres is designed to ensure the sliding constraints freely rotate and the gravity center maintains the piezo-MM and sliding cell in the vertical direction under arbitrary ocean waves. Postbuckling response (i.e., high-frequency vibration) is obtained due to the sliding constraints, and thus, the electrical power is generated from the piezoelectric strips. The bi-walled piezo-MMs are designed in the gyro-structure, and thus, the reported MM-GPENG can be activated under ocean waves in arbitrary directions. The piezo-MM plate length and width are denoted as *L* and *b*, respectively, and the corrugation pattern consists of the diameter *D*, rib width *W*, and height *h*_hex_. Note that the constraint gap of the sliding cell is *g* and the thicknesses of the MM and piezo layers are *t* and *t*_p_, respectively. Figure 1c illustrates the design principle of the energy harvester under ocean motions. The fluctuation of ocean surface provides periodically vibrated and quasi-static ocean waves, which can be deployed to trigger piezo-MM for electrical power via the postbuckling response design in the gyro-structure units. By assembling the units into the energy harvesting net, the energy output of the MM-GPENG can be increased and controlled for different applications.

**Figure 2:**
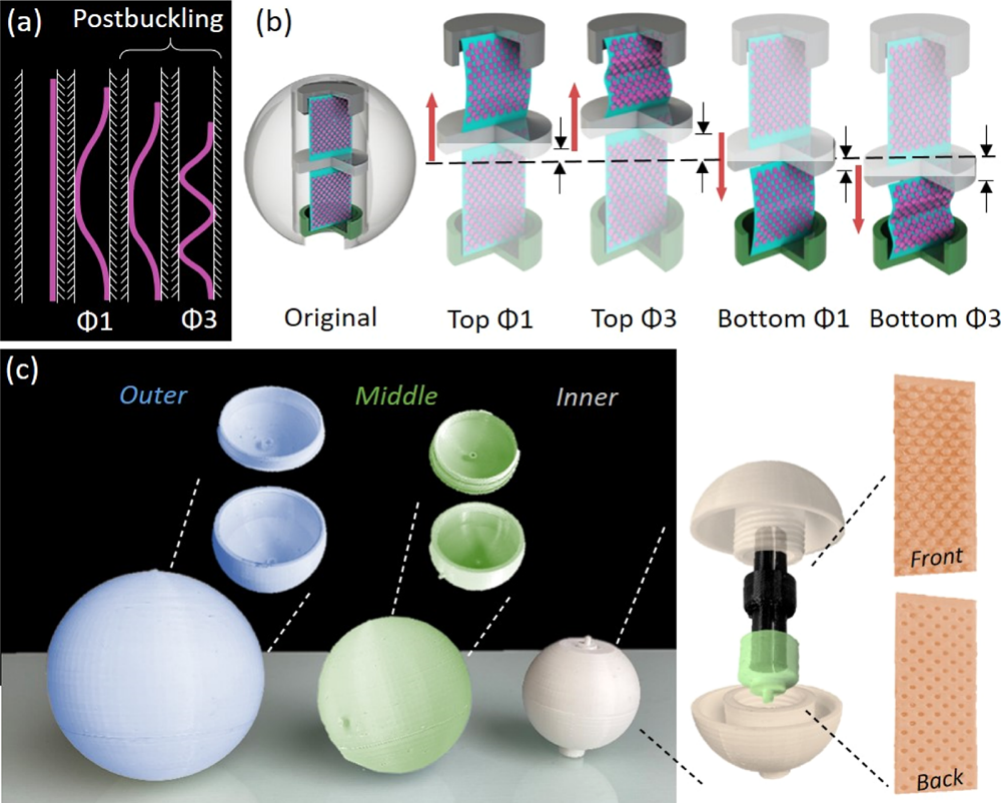
Principle and fabrication of the MM-GPENG. (a) Postbuckling process of the piezo-MM plates triggered by the ocean waves under the sliding constraints. (b) Top and bottom piezo-MM plates deformed to the first and third postbuckling modes due to the axial displacement. (c) Fabrication and assembly of the MM-GPENG using the 3D printing technique.

Figure 2a shows the postbuckling response of a piezo-MM plate between the bilateral constraints. The initially straight plate buckles to the first buckling mode (Φ1) until it touches the constraint with the point contact. With the increasing axial displacement, the point contact between the buckled plate and the constraints grows to the line contact. With further increasing the axial displacement, the line contact meets the limit state and then snaps into the third buckling mode (Φ3). Through the buckling mode transitions such as the snap-throughs between Φ1−Φ3 or Φ3−Φ5, the high-speed and high-acceleration local response are obtained. Therefore, the piezoelectric material attached to the MM plates is effectively triggered. The higher buckling modes after the first buckling mode (e.g., Φ3) are typically referred to as the postbuckling response. Figure 2b displays the postbuckling process of the piezo-MM plates under the axial displacement caused by the ocean waves. In particular, the top and bottom piezo-MM plates are deformed to the first and third postbuckling modes due to the bilateral constraints of the sliding cell. According to the design principle, the piezo-MM plates are the only components that are deformed under the cyclic loading in the ocean. As a consequence, it is important to ensure that the plates are deformed in the linear elastic domain such that the functionality of the MM-GPENG can be remained. In the current design, the piezo-MM plates are deformed in the linear elastic domain due to the following two reasons. First of all, the plates are placed between the bilateral constraints. The gap between the constraints is much smaller than the length of the plates (e.g., *g*/*L* = 0.055 in Table 1). Therefore, the plates are buckled to higher buckling modes (i.e., postbuckling response) under the cyclic loading, instead of being largely deformed in the first mode. In other words, the piezo-MM plates are postbuckled in the small deformation. Second, the hexagonal corrugation in the plates leads to complete recovery after large deformations (i.e., the plates are remained in the linear elastic domain). Our previous study has investigated and concluded the good recoverability of the MM plates at the multiscale level.^50^

**Table 1:** Geometric and Material Properties and Element Size of the Piezo-MM

geo. property	overall (mm)	length L	55
		width *b*	15
		MM thickness *t*	0.5
		piezo thickness tp	0.5
		height hhex	2
		walls gap g	3
	walls (mm)	diameter D	4
		rib width W	2
material property	piezo-MM	*E*_p_ (GPa)	3.5
		*E*_MM_ (GPa)	5.5
		*v*_p_	0.2
	walls	*E*_c_	Rigid
mesh	plates	*l*_p_	0.5
	walls	*l*_c_	
loading	shape	sinusoidal	
	amplitude (mm)	1.5 and 2.5	
	period (s)	5 and 10	

Figure 2c presents the fabrication of the MM-GPENG using the 3D printing technique at the millimeter scale. The corrugated MMs were fabricated by polylactic acid (PLA) using the additive manufacturing technique, and the PVDF strips were bonded to the MM plates, which are grouped to the inner, middle, and outer spherical gyro-structures. Different piezoelectric materials have been developed to generate electrical power from the environment in different working scenarios. For example, lead zirconate titanate (PZT) is typically used to attach to rigid surfaces without obvious deformation because the PZT is rigid and brittle. PVDF, on the other hand, is flexible such that it can be used to undergo certain mechanical deformation. In this study, the piezo-MM plates are designed to buckle and postbuckle between the bilateral constraints, and therefore, PVDF is used as the energy harvesting material.

The contact friction between the plates and bilateral constraints plays a critical role in the MM-GPENG. For example, the existing study has reported the postbuckling-enabled damper based on the contact friction between the plates and bilateral constraints.^49^ In this study, however, we aim to completely convert the ocean waves into mechanical energy in the piezo-metaplates. Therefore, it is of necessity to eliminate the contact friction. To reduce the possible friction in the MM-GPENG, the lubrication was coated on the bilateral constraints in the experiments. In addition, as long as the primary characteristics of the plate-like MM are maintained, the MM-GPENG can be efficiently triggered to generate electrical power at the multiscale level. The previous study has reported the possibility of using the MM plates to generate power at the nanoscale level.^50^ In particular, to scale down the piezo-metaplates and create electrical power at the micro/nanoscale level, it is significant to maintain the geometric ratios (e.g., the hexagonal pattern of the plates and the distance to the bilateral constraints).

### 2.2 Theoretical Modeling of the MM-GPENG

#### 2.2.1 Mechanical Modeling

**Figure 3:**
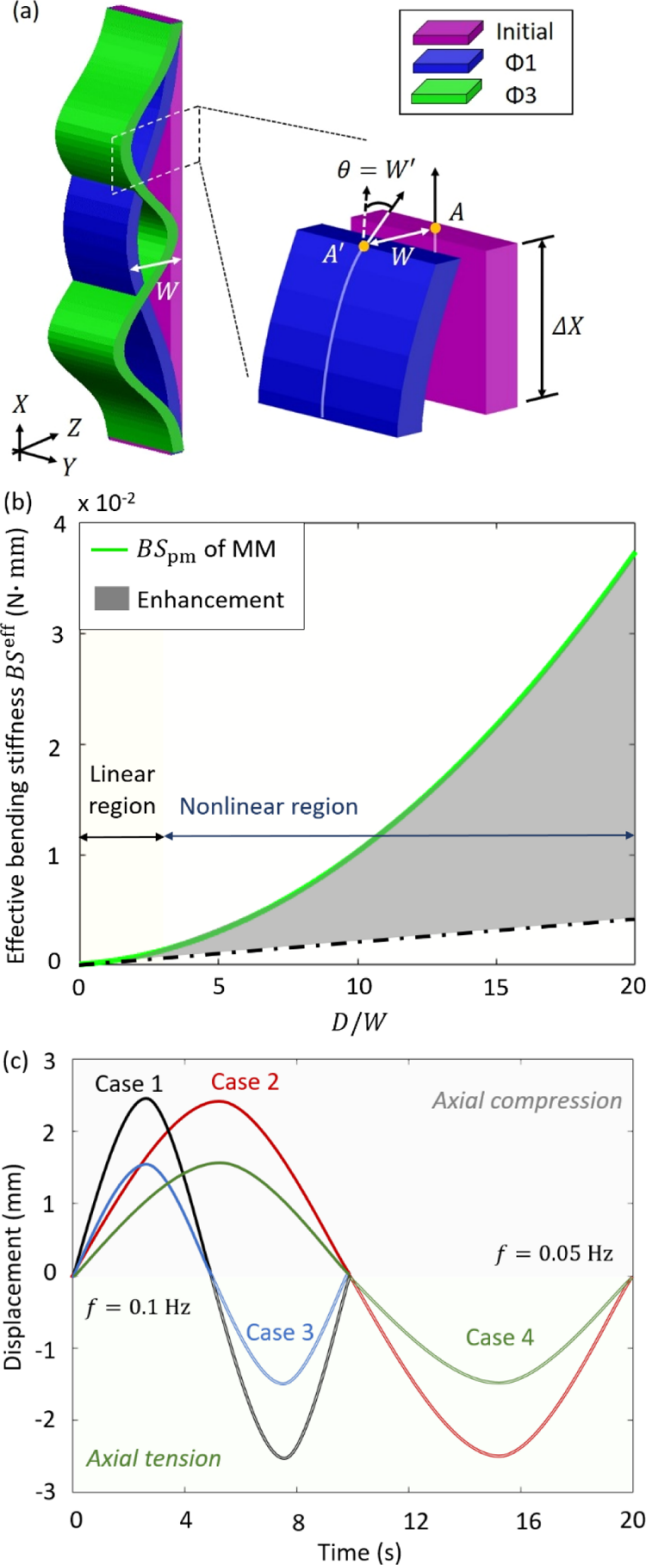
Theoretical modeling and results. (a) Illustration of the postbuckling response and deformation analysis of an arbitrary segment in the deformed piezo-MM. (b) Comparison of the effective bending stiffnesses between the MM plates and the planar sheets comprising the same geometric properties (i.e., length, width, and thickness). (c) Axial displacement history of the quasi-static excitations caused by ocean waves.

In this section, a theoretical model is developed to investigate the electrical power generated by the MM-GPENG under the ocean motions. The theoretical model particularly studies the improvement of the harvested power due to the mechanical characteristics of the corrugated piezo-MM. Figure 3a illustrates the postbuckling response (i.e., mode 1 and mode 3) of the piezo-MM subjected to the ocean-induced axial force. The transverse displacement and rotation angle of the MM plates are denoted as *W* and θ, respectively, and we define d*W*(*X*)/d*X* = θ(*X*).

According to our previous studies, the bending stiffness enhancement factor of the alumina MM with hexagonal corrugation can be written as^50^
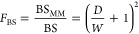
1where BS_MM_ and BS denote the effective bending stiffnesses of the corrugated plates and planar sheets comprising the same geometries as the MM, respectively. Therefore, the effective bending stiffness of the piezo-MM is defined as
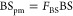
2where BS = EI is the bending stiffness of the planar sheets attached by piezoelectric strips. Note that the corrugated MM is simplified to the planar beam using the effective bending stiffness in eq 2. Figure 3b presents the effective bending stiffnesses of the piezo-MM plates with and without the hexagonal corrugation. Compared to the planar sheets with linear bending stiffness, the mechanical characteristics of the piezo-MM are significantly enhanced due to the hexagonal corrugation. Due to the large aspect ratio (i.e., the thickness-to-width ratio), Poisson’s ratio of the MM is omittable (i.e., *v* = 0).

Due to the constraints, the beams are deflected in the moderately small manner. Therefore, the nondimensional governing equation of the piezo-MM subjected to the ocean motions is
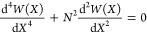
3aand
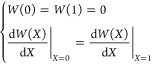
3bwhere the ocean-induced axial compressive force *N* is
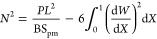
4and the nondimensional factors are *X* = *x*/*L* and *W* = *w*(*XL*)/*g*. Because the loading edge is free to slide in the longitudinal direction and the bottom edge is clamped, the axial displacement is applied to the plate top in the theoretical model. In this study, a sinusoidal displacement function is particularly used to formulate the ocean waves, as shown in Figure 3c
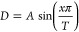
5where the amplitude and time period were *A* = 1.5 and 2.5 mm and *T* = 10 and 20 s, respectively. In particular, the displacement is defined as axial tension and compression such that the top and bottom MM-piezo plates can be triggered (Figure 2a). The general solution for eqs 3a and 3b can be expressed as
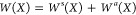
6where the symmetric and asymmetric mode shapes are given as, respectively
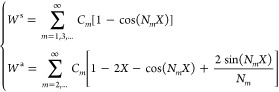
7

Note that *N*_*m*_ = (*m* + 1)π and *N*_*m*_ = 2.86π, 4.92π, 6.94π, 8.95π,... represent the symmetric and asymmetric buckling modes of the MM, respectively.

To determine the unknown coefficients *C*_*m*_ in eq 7, an energy method is proposed to minimize the total energy of the piezo-MM between the sliding constraints (Figure 1a). Because the sliding constraints are activated by typically frictionless, quasi-static ocean motions, the total energy of the piezo-MM at any equilibrium state can be assumed to be the same as the total potential energy (i.e., the kinetic energy is negligible).^49^ The normalized total potential energy of the slidingly constrained piezo-MM is given as
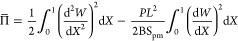
8where the nondimensional factor is: . Taking eq 7 into eq 8, we obtain the total energy of the postbuckled piezo-MM as
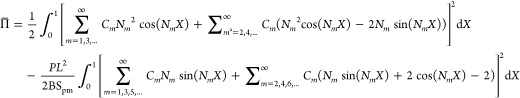
9

Next, the total energy is used to determine *C*_*m*_ in eq 9. Note that the sliding constraints in the MM-GPENG can be mathematically modeled as the constraints imposed on the transverse deflection of the plates. The deflection of the piezo-MM should always be bounded by the distance between the sliding walls. As a consequence, the total energy is minimized with respect to the unknown coefficients *C*_*m*_ between the sliding constraints as
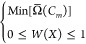
10

Because the objective function of the total energy is nonlinear, the Nelder–Mead algorithm is used in this study to numerically solve *C*_*m*_ in the minimization problem. Substituting the factors into eq 6, the postbuckling response of the piezo-MM can be obtained.

#### 2.2.2 Electrical Modeling

Because the corrugated piezo-MM plates have an extremely large aspect ratio (i.e., *b*/*t* ≫ 1), the axial shortening of the plate is negligible (i.e., piezo-MM is inextensible) and the axial displacement only resulted in the postbuckling-induced transverse displacement. The axial strain of the postbuckled piezo-MM can be written as^51^
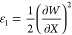
11and hence, the axial stress is
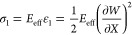
12where effective Young’s modulus can be obtained based on eq 1 as *E*_eff_ = *F*_BS_*E*, and the subscription denotes the loading direction of the ocean motions.

We formulate the piezo-MM using the Euler–Bernoulli beam theory, which only takes into account the axial stress of the piezo (i.e., the rest of the stress components are omitted).^52,53^ The transverse electric displacement of the piezo-MM can be written as
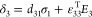
13where *d*_31_, ε_33_^T^, and *E*_3_ are the piezo strain tensor, permittivity tensor, and electric field in the transverse direction, respectively.

The total electric charge *Q* is obtained as the integral of the transverse electric displacement over the entire area of the piezo plate *A* = *Lb*.
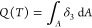
14and the voltage across the load resistance *R*_L_ is obtained as
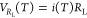
15where *i*(*T*) represents the current in the closed-circuit condition as *i* = d*Q*/d*T*. Taking eqs 13 and 14 into eq 15, we have
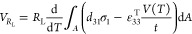
16where *E*_3_ = −*V*(*T*)/*t* describes the voltage in the transverse electrical field. Equation 15 can be rewritten as
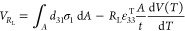
17

The electrical power generated by the piezo-MM under ocean motions is
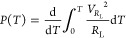
18

Equation 18 can be rewritten as
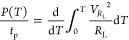
19

Because the voltage across the equivalent capacitance of the piezoelectric patch is the same as the voltage across the resistive load, that is, *V* = *V*_*R*_L__, the generated electrical energy is eventually obtained as
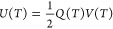
20

### 2.3 Numerical Modeling of the MM-GPENG

#### 2.3.1 Numerical Modeling

**Figure 4:**
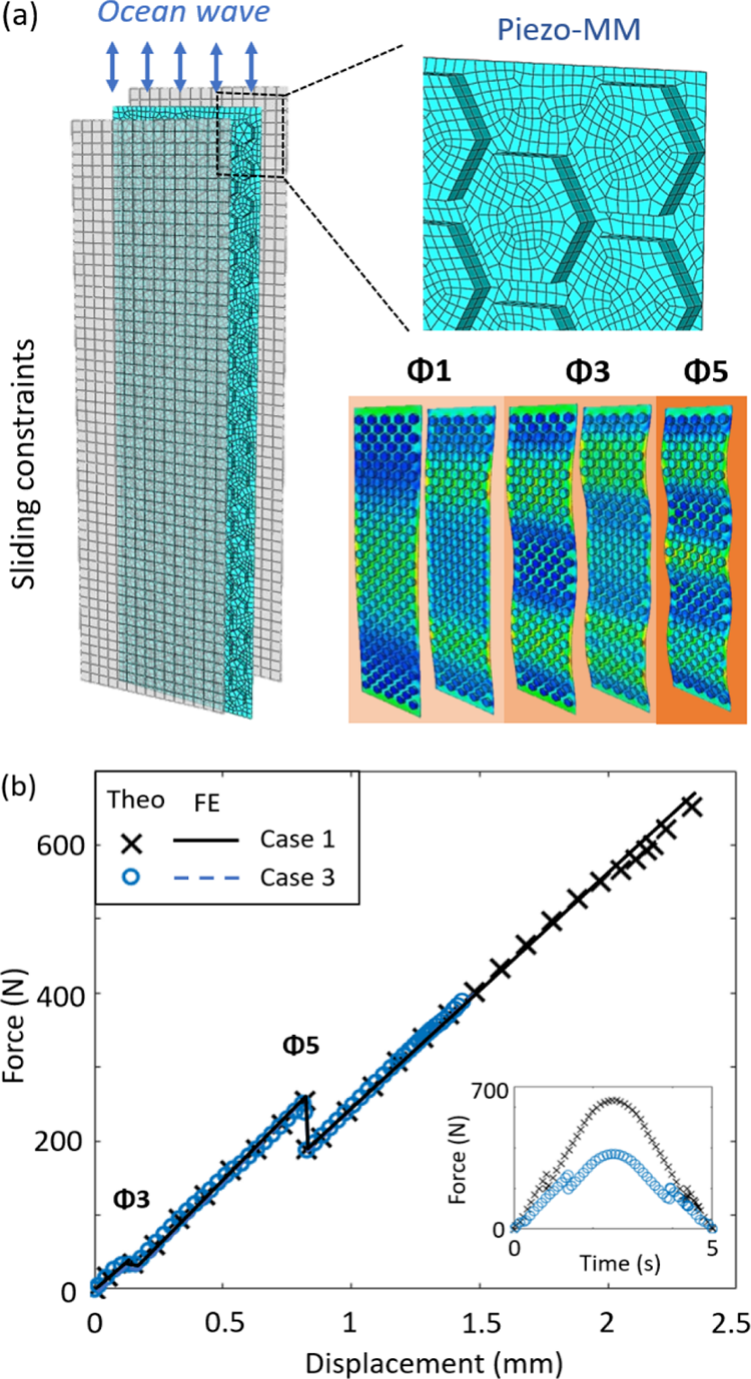
Numerical modeling and comparison. (a) Numerical modeling and the deformation configurations of the piezo-MM in the buckling modes Φ1, Φ3, and Φ5. (b) Comparison of the force–displacement response between the theoretical and numerical results for the piezo-MM plates under the loading conditions case 1 and case 3 defined in Figure 3c.

Numerical simulations are conducted to investigate the electrical output of the MM-GPENG using Abaqus v6.14-1. Figure 4a shows the numerical modeling of the corrugated MM subjected to the bilateral confinements. To obtain the postbuckling response of the piezo-MM, two types of calculation algorithms are particularly used, that is, linear perturbation/buckle for the buckling analysis and dynamic implicit for the postbuckling analysis. Buckling imperfection is considered by modifying the input files.^49^ A shell element (S4R) is used in the FE model, and the contact interaction is defined to simulate the constraints of the sliding cell on the postbuckled piezo-MM plates. To simplify the sliding boundary conditions in the FE model, the bilateral constraints are fixed, and the ocean motions applied to the top edge of the piezo-MM are given in eq 5. The time periods used in the ocean motion function are 5 and 10 s and the amplitudes are 1.5 and 2.5 mm. The geometric and material properties of the FE models are listed in Table 1.

#### 2.3.2 Comparison between the Theoretical and Numerical Results

Figure 4b compares the theoretical and numerical results to validate the accuracy of the theoretical model. The tensile and compressive force–displacement relations are obtained under the ocean wave excitations (i.e., the loading conditions case 1 and case 3 defined in Figure 3c). It can be seen that the reported theoretical model accurately predicts the buckling mode transitions of the bi-walled piezo-MM plates. The time history of the reaction force is also provided. More interestingly, the deformation resistance (i.e., the slope) is maintained the same before and after the postbuckling mode transitions, which demonstrates the repeatability of the reported MM-GPENG. In addition, the repeatability is critical in maintaining the functionality of the MM-GPENG under ocean motions over a relatively long time period.

### 2.4 Generated Electrical Power of the MM-GPENG

**Figure 5:**
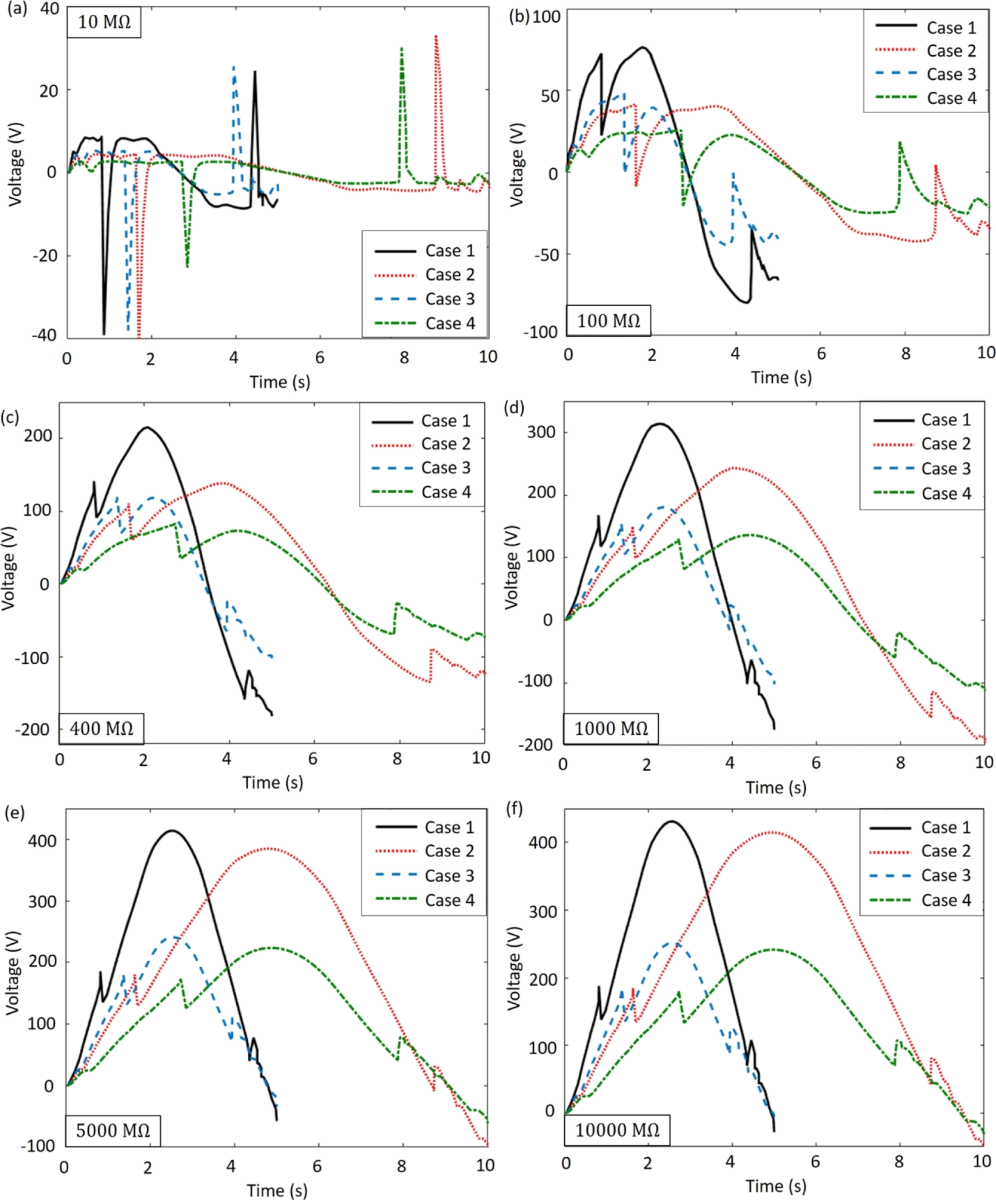
Comparison of the closed-circuit voltages. The generated voltages under the load resistance of (a) *R*_L_ = 10 MΩ, (b) *R*_L_ = 100 MΩ, (c) *R*_L_ = 400 MΩ, (d) *R*_L_ = 1000 MΩ, (e) *R*_L_ = 5000 MΩ, and (f) *R*_L_ = 10,000 MΩ (the four loading conditions defined in Figure 3c are used in all the cases).

In this section, the generated electrical power of the MM-GEPNG is investigated using the numerical models. Figure 5 presents the closed-circuit voltage generated using the energy harvesting technique under the four loading conditions. In particular, the generated voltages are provided under the load resistance of *R*_L_ = 10–10,000 MΩ in Figure 5a–f, respectively. It can be seen that the obtained voltages are increased with respect to the load resistance. However, the highest influence of the load resistance is obtained at around *R*_L_ = 500 MΩ (i.e., the increasing rate of the voltage is reduced when the load resistance is larger than 500 MΩ).

**Figure 6:**
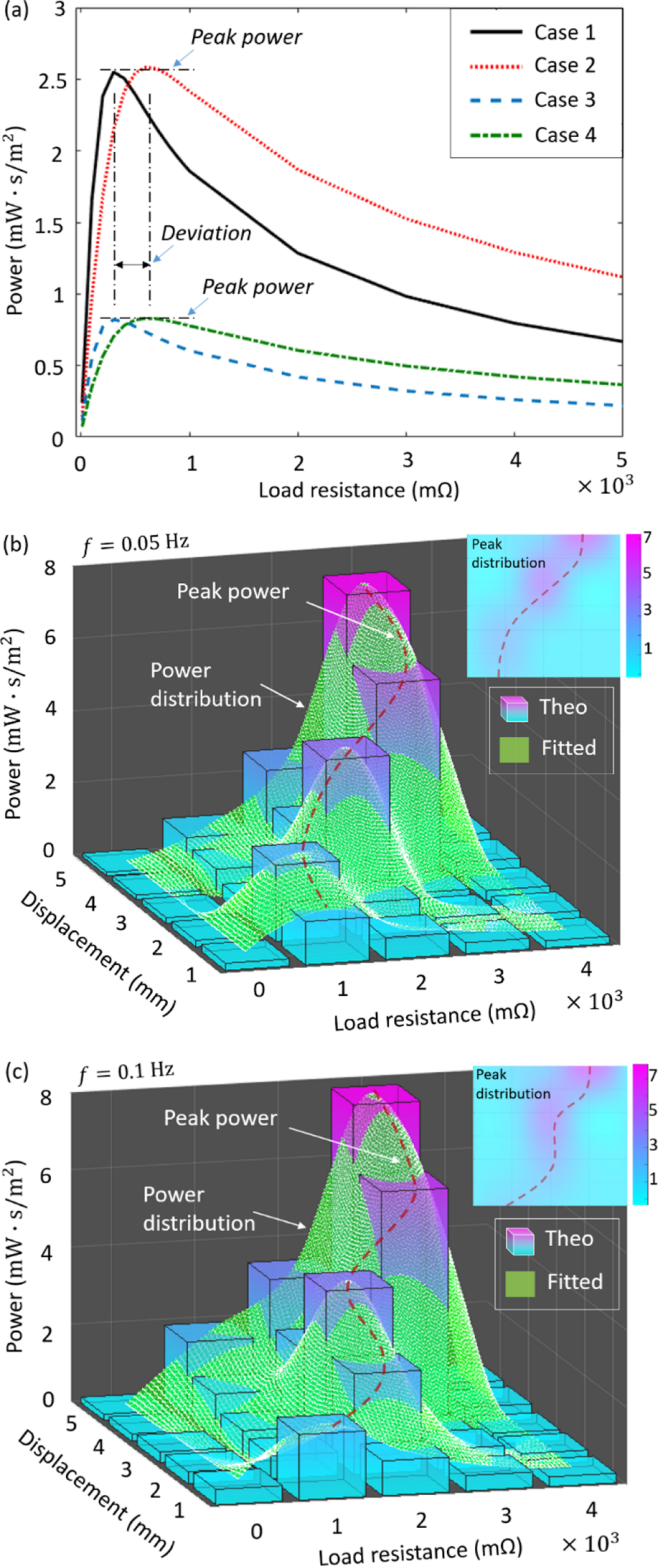
Electrical power of the MM-GPENG. (a) Generated electrical power in terms of the load resistance. Numerical results of the power under different loading conditions and load resistances for (b) *f* = 0.05 Hz in case 1 and case 3 and (c) *f* = 0.1 Hz in case 2 and case 4.

Figure 6a displays the generated power of the MM-GPENG with respect to the load resistance, which is in coincidence with the findings in Figure 5 showing that the peak power is obtained with the load resistance of *R*_L_ = 500 MΩ. Comparing the output power under the loading conditions case 1 and case 2, we conclude that the loading time period shifts the power history while maintaining the values to be the same. On the contrary, the loading amplitude only affects the values of the power. Figure 6b,c shows the electrical power with axial displacement and load resistance at the loading frequencies of *f* = 0.1 and 0.05 Hz, respectively. Nonlinear fitting is applied to obtain the distribution of the electrical power using the theoretical data. The shifting of the peak power indicates that the displacement and load resistance critically affect the generated energy.

## 3 Discussion

In this section, we investigate the programmability of the MM-GPENG to optimize the electrical power using the geometric and material approaches. In particular, the geometric method is conducted by changing the corrugation pattern of the MM plates (Figure 1a) from hexagonal to rectangular and cylindrical, and the material method is conducted by varying the material properties of the hexagonal MM from isotropic to anisotropic.

### 3.1 Geometric Method of Changing the Corrugation Pattern of the MM Plates

**Figure 7:**
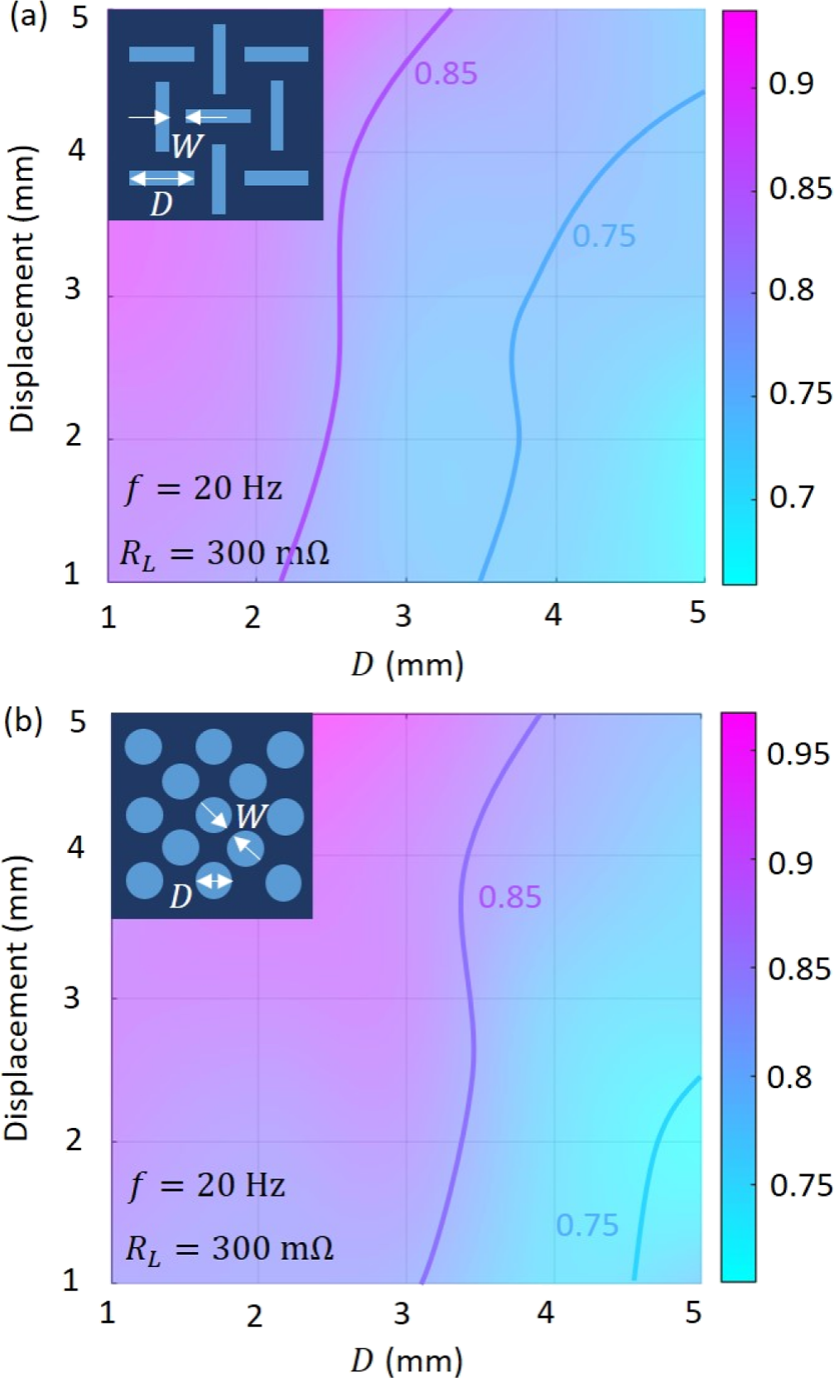
Programmability of the MM-GPENG using the geometric method. Ratios of the electrical power between the hexagonal MM-GPENG and the MM plates with (a) rectangular and (b) cylindrical corrugation patterns. (*f* = 0.05 Hz and *R*_L_ = 500 MΩ in both the cases).

The postbuckling response, in particular the snap-through, is critical to the electrical power of the MM-GPENG. As a consequence, the geometric consideration is carried out to investigate the influence of the MM plates’ corrugation pattern on the generated energy. Figure 7 shows the power ratio of the MM-GPENG with different corrugation patterns.

Bending performance of the piezo-MM plates (e.g., bending stiffness) plays a significant role in maneuvering the mechanical response of the plates, which therefore affects the energy generation of the MM-GPENG. According to the previous studies on the mechanical characteristics of the MM plates, we have found that the pattern length *D* affects the bending performance of the plates more significantly than that of the width *W*.^50^ As a consequence, this study maximizes the generated energy with respect to *D*. Figure 7a presents the energy ratio between the hexagonal and rectangular MM-GPENG. The pattern length *D* is varied, and the pattern width *W* is fixed as . Note that the excitation frequency and load resistance are fixed as *f* = 0.05 Hz and *R*_L_ = 500 MΩ, respectively. It can be seen from the ratio distributions that the influence of the corrugation pattern is enhanced when *D* is increased, which can be explained by the fact that the rectangular pattern tends to become more sparse compared to the hexagonal pattern with the same *D*. Figure 7b displays the energy ratio between the hexagonal and cylindrical MM-GPENG. The cylindrical pattern is likely to affect the generated energy that is less significant than that of the rectangular pattern.

### 3.2 Material Method of Changing the Material Properties of the Hexagonal MM Plates

The electrical power is studied in terms of the material properties of the MM plates with hexagonal corrugation. In particular, Young’s modulus of the MM plates is expanded from isotropic (i.e., PLA) to bi-layered anisotropic [i.e., PLA and acrylonitrile butadiene styrene (ABS)]. The bimaterial property is defined with the plate length as
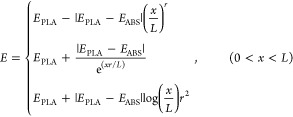
21where *r* = *V*_PLA_/*V*_ABS_ describes the variation in the volume fraction, and *E*_ABS_ is defined as Young’s moduli of PLA and ABS.

**Figure 8:**
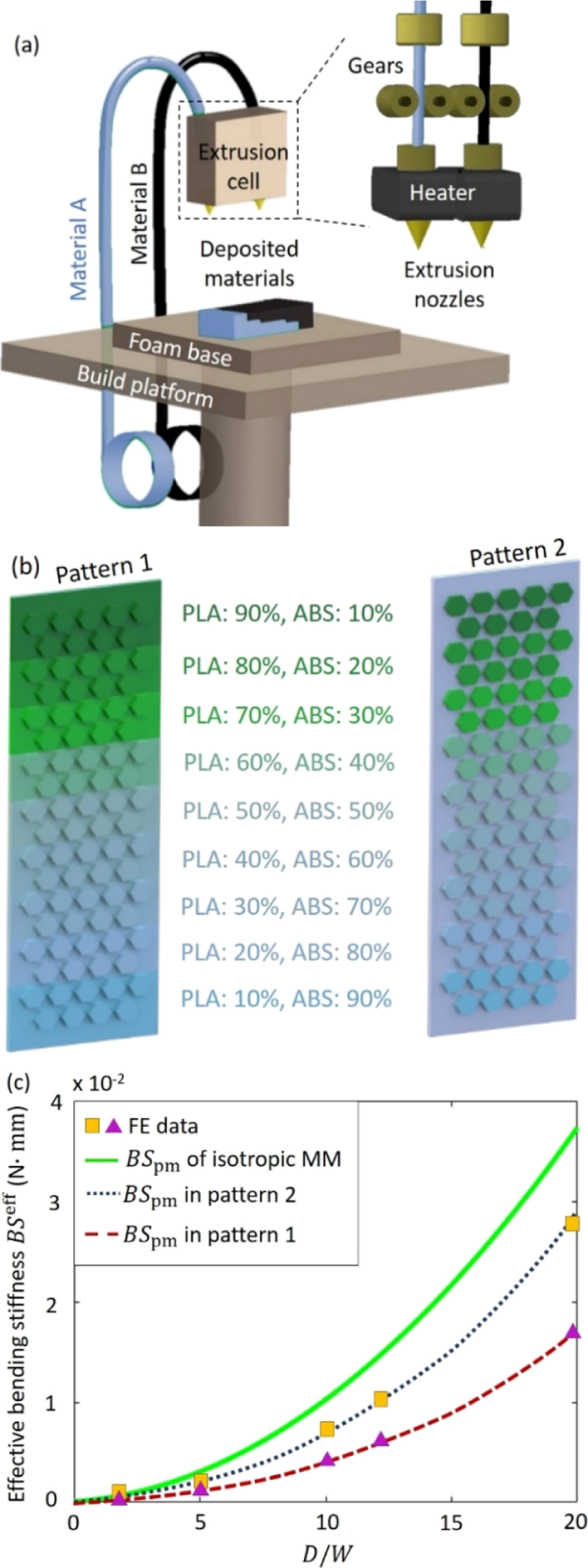
3D additive manufacturing of the anisotropic MM plates in the MM-GPENG. (a) Illustration of the bimaterial 3D printer to manufacture the anisotropic MM plates. (b) Distribution patterns of the anisotropic MM plates designed with hexagonal corrugation. (c) Comparison of the effective bending stiffnesses between the isotropic and bimaterial MM plates in pattern 1 and pattern 2.

Next, the anisotropic plates were fabricated using the 3D additive manufacturing technique. The Ultimaker-S3 dual nozzle printer, implementing the fused filament fabrication technology, was particularly used to manufacture the bimaterial MM. Figure 8a illustrates the components and printing principle of the dual nozzle 3D printing. The polymer filaments were fed and heated through the extrusion cell to convert the materials states from hard to soft, which were then printed on the platform, following the signed corrugation pattern. The extrusion cell was designed with two brass nozzles with a diameter of 0.4 mm, which can be freely moved in the *x*–*y* plane, and the platform can be moved in the *z* direction layer by layer with the tolerance of approximately 0.06 mm until the predefined pattern was completed. The material properties of the PLA and ABS are listed in Table 2.

**Table 2:** Materials Properties of the Anisotropic MM Beams

	PLA	nylon	ABS
density (g/cm^3^)	1.24	1.14	1.1
Young’s modulus (GPa)	3.47	0.889	2.07
elongation at break (%)	5.2	210	4.8
ratio of flexural modulus-to-tensile modulus	1.34	0.8	1.28
filament length/weight (mm/g)	126.67	137.33	142.67

Figure 8b demonstrates the two distribution patterns of the PLA/ABS bimaterial. In pattern 1, the bimaterial is varied for both the face sheet and hexagonal corrugation and pattern 2 only changes the corrugation while maintaining face sheet the same material (i.e., PLA). Figure 8c compares the effective bending stiffnesses between the isotropic and anisotropic MM plates in the two bimaterial patterns. Taking eq 21 into eq 2, the varied bending stiffness can be written as
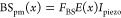
22

According to the equilibrium conditions of the local moment, the governing equation of the anisotropic MM plates can be obtained by expanding eqs 3a and 3b to
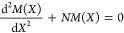
23where  and the normalized transverse deflection *W*(*X*) are used to define the curvature of the beam *M*(*X*), as
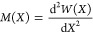
24where *X* = *x*/*L* and *W*(*X*) = *ŵ*(*XL*)/*h*. The general solution of eq 23 can be expressed as
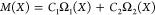
25where *C*_1_ and *C*_2_ are the unknown integral constants, and Ω_1_(*X*) and Ω_2_(*X*) represent the linearly independent special solutions for different cross-section area configurations, respectively. Integrating the curvature *M*(*X*) leads to the general solution for the anisotropic MM plates as
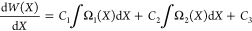
26
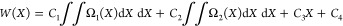
27where the constants *C*_*i*_ (*i* = 1,...,4) can be determined by the fixed–fixed boundary conditions.

**Figure 9:**
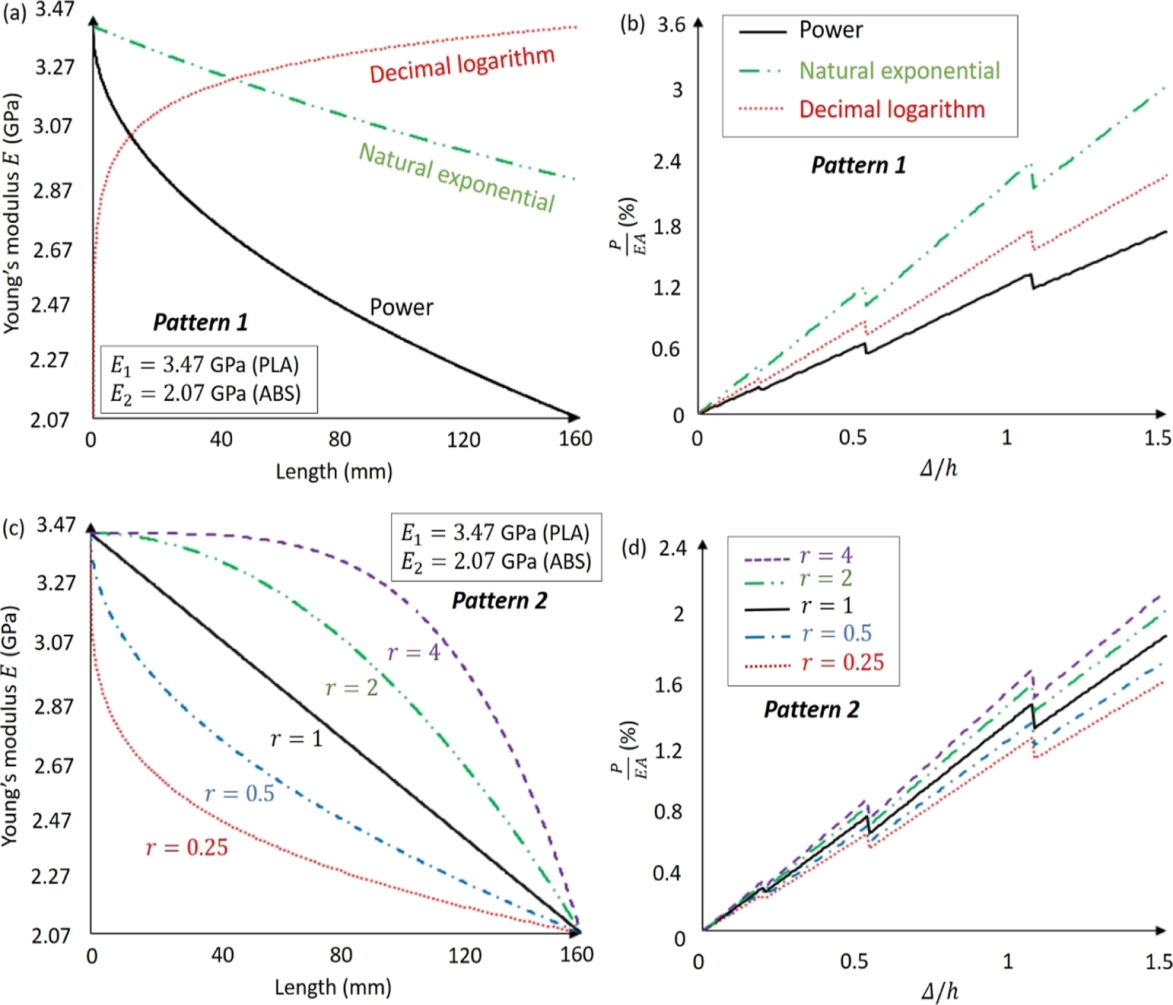
Material properties and normalized force–displacement relations of the bimaterial MM. (a) Young’s modulus variations and (b) normalized force–displacement relations of the bimaterial MM plates in pattern 1. (c) Young’s modulus variations and (d) normalized force–displacement relations of the bimaterial MM plates in pattern 2.

In the same manner as the theoretical modeling mentioned in Section 3.2, the postbuckling responses for the MM plates with anisotropic material properties are analyzed using the energy method. The Young’s modulus and force-displacement relations of the bimaterial MM are given in Figure 9. The total potential energy of the deformed plates can be written as the summation of the bending strain energy *u*_b_, compressive strain energy *u*_c_, and energy of external work *u*_p_, which can be expressed in terms of the unknown coefficients in the buckling mode shapes as 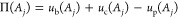
28where the energy components are
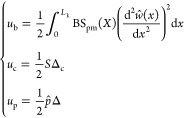
29

Note that Δ_c_ refers to the axial compressive deformation, which can be written as
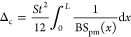
30

Considering the gradually increasing displacement applied to the MM plates, the axial compressive force *S* can be expressed as
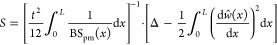
31where Δ is the variation of the beam length that is given as
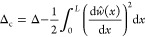
32

Therefore, the total potential energy can be written as
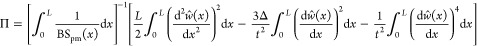
33

Substituting the boundary conditions and eq 6 into eq 33, the total potential energy can be expressed as
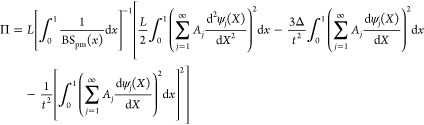
34where *j* = 1,...,∞. Substituting eq 34 into eq 10, the postbuckling response of the anisotropic MM can be determined.

**Figure 10:**
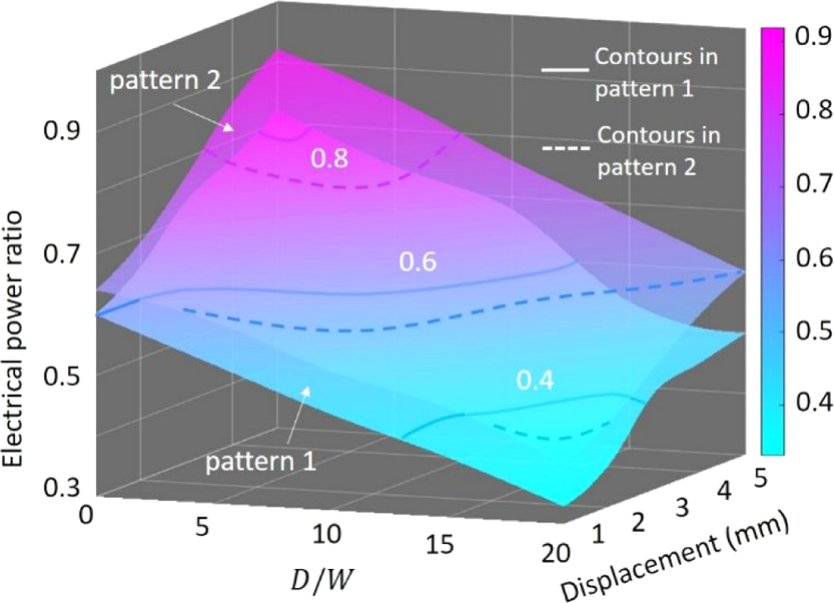
Electrical power ratio. Comparison of the electrical power generated by MM-GPENG designed with the isotropic and anisotropic piezo-MM in pattern 1 and pattern 2 (the electrical power ratio is defined as power_iso_/power_aniso_).

Figure 10 shows the ratios of the electrical power generated by the MM-GPENG with the isotropic and bimaterial piezo-MM plates in patterns 1 and 2. In particular, the results are presented in terms of the power ratio as power_iso_/power_aniso_. It can be seen that the bimaterial distribution critically affects the output power because the mechanical response of the MM plates is influenced. The obtained energy ratio is in coincidence with the results given in Figure 8c. More importantly, the programmable MM can be used to effectively tune the harvested electrical energy in the MM-GPENG. As a consequence, the reported MM-GPENG can be applied in various fields for different purposes.

## 4 Conclusions

This study reported the MM-enabled piezoelectric nanogenerators in gyro-structures for energy harvesting under quasi-static excitations (i.e., <1 Hz) in the ocean environments. The plate-like MMs designed with a hexagonal corrugation pattern were placed between the sliding constraints, which were assembled in the gyro-structure to trigger the postbuckling response for energy harvesting under low-frequency excitations. The MM plates were fabricated using a 3D additive manufacturing technique, and the PVDF strips were attached to obtain the corrugated piezo-MM. The theoretical and numerical models were developed to predict the output power, and satisfactory agreements were obtained in the validation. The energy harvesting performance of the MM-GPENG was optimized using the geometric method (i.e., varying the corrugation pattern) and material method (i.e., changing Young’s modulus to anisotropic). Taking the reported MM-GPENG as the energy harvesting unit and connecting many of those units into an energy harvesting net, the output electrical power is enlarged. Future work can be conducted to investigate the performance of the energy harvesting net. Although the output power is expected to be affected by the number of the MM-GPENG units, the external excitations also critically affect the generated electrical power of the energy harvesting units, especially in a relatively large energy harvesting net with many units. The MM-GPENGs are considered as a potential green solution for energy issues in other fields.
